# Cancer metastasis through the lymphatic versus blood vessels

**DOI:** 10.1007/s10585-024-10288-0

**Published:** 2024-06-28

**Authors:** Stanley P. Leong, Marlys H. Witte

**Affiliations:** 1 California Pacific Medical Center and Research Institute, University of California School of Medicine, San Francisco, USA; 2 Department of Surgery, Neurosurgery and Pediatrics, University of Arizona College of Medicine-Tucson, Tucson, AZ USA

**Keywords:** Cancer metastasis, Lymphatic and blood vessels

## Abstract

Whether cancer cells metastasize from the primary site to the distant sites via the lymphatic vessels or the blood vessels directly into the circulation is still under intense study. In this review article, we follow the journey of cancer cells metastasizing to the sentinel lymph nodes and beyond to the distant sites. We emphasize cancer heterogeneity and microenvironment as major determinants of cancer metastasis. Multiple molecules have been found to be associated with the complicated process of metastasis. Based on the large sentinel lymph node data, it is reasonable to conclude that cancer cells may metastasize through the blood vessels in some cases but in most cases, they use the sentinel lymph nodes as the major gateway to enter the circulation to distant sites.

## Introduction

**Stanley P. Leong**

The hallmarks of cancer of Hanahan and Weinberg include proliferative receptor signaling, evading growth suppressors, resisting cell death, cell immortality, inducing angiogenesis, acquiring the ability to invade and metastasize, increased metabolism, increased genome instability and mutation, avoiding immune destruction and promoting inflammation [1]. Cancer is not a uniform disease but consists of many different types that can vary significantly between patients, as well as between primary cancer sites and their metastases. This heterogeneity arises from genetic mutations [2, 3] and epigenetic changes [4] that alter the DNA sequence or the regulation of genes without changing the DNA sequence respectively. These changes can lead to the development of various clones within a cancer population, each with potentially different characteristics. Within the cancer microenvironment, cancer cells undergo a process similar to Darwinian “natural selection” [5], where cells with advantageous mutations that allow them to grow, evade the immune system, or resist therapy. Therefore, they are more likely to survive and proliferate. This selection process can lead to the emergence of more aggressive and therapy-resistant cancer clones. Metastasis depends on the interaction between the ‘seed’ (cancer cells) and the ‘soil’ (the target organ’s microenvironment) according to Paget’ seed and soil hypothesis [6]. Not all cancer cells (seeds) can form metastases in any organ (soil); they require a compatible environment that supports their growth and survival. Cancer cells can metastasize from the primary site to distant organs through different routes. They can invade local lymphatic vessels and travel to regional lymph nodes or sentinel lymph nodes (SLNs), enter the bloodstream to reach distant organs, or directly enter the blood vessels at the primary cancer site. The choice of pathways can influence the site of metastasis and the clinical approach to treatment. The process by which cancer cells evolve, metastasize and establish new sites in distant organs is complex and involves numerous cellular and molecular mechanisms. These include changes that allow cancer cells to detach from the primary site, invade surrounding tissues, survive in the circulation, exit the bloodstream, and grow in a new microenvironment. Angiogenesis is a critical process for cancer development and metastasis, providing the cancer cells with nutrients and oxygen and allowing cancer cells to enter the bloodstream and lymphatic system, leading to metastasis to other parts of the body. Key factors like Vascular Endothelial Growth Factor (VEGF) [7] and Hepatocyte Growth Factor (HGF) [8] play significant roles in promoting angiogenesis, making them important targets for cancer therapy. Markers such as Lymphatic Vessel Endothelial Hyaluronan Receptor-1 (LYVE-1) and Vascular Endothelial Growth Factor Receptor-3 (VEGFR-3) are specifically involved in lymphangiogenesis. LYVE-1 is a marker for lymphatic endothelial cells, and VEGFR-3 is primarily expressed in the endothelial cells of the lymphatic vessels [7]. These markers are crucial for understanding the mechanisms behind angiogenesis and lymphangiogenesis and their role in cancer metastasis. Understanding these mechanisms is crucial for developing targeted therapies to prevent and treat metastases.

To date, the molecular mechanisms of cancer metastasis are under intense study. A review of lymphatic systemomics and cancer will be appropriate to set the stage for cancer cells to traverse the lymphatic system. The identification of molecules to facilitate cancer metastasis may allow us to use as biomarkers to describe and predict cancer metastasis. Perhaps, these molecules may be blocked to impede the process of metastasis.

### Lymphatic systemomics and cancer

**Marlys H Witte**

Since the discovery of lymphatic circulation by Gaspar Aselli of Padua in 1627 [9], the connection between the lymphatic system and cancer was not established until Virchow’s demonstration that cancers were associated with proliferating abnormal cells (“cellular theory”) in 1860 [10]. The relationship between the lymphatic system and cancer growth became more firmly established (Table 1). Cancer metastasis to enlarged regional lymph nodes continued to be recognized leading to progressively more radical lymphadenectomies (operations) by Halsted [11] to remove and interrupt the pathways of metastasis through the lymphatic vessels draining the primary cancer.

The lymphatic system could no longer be viewed as “lymph nodes held together by strings” but instead was an integrated system of lymphatic vessels, circulating lymph fluid, lymph nodes, and trafficking lymphocytes. Lymphatic “systemomics” was born, i.e., the lymphatic system as a distinctive vasculature, a circulation passing through lymph nodes and extending from the interstitium to the entry of central lymph collectors into the bloodstream, a route of transport of abnormal particles and microbes from the external environment, and the immune network system itself including the lymphoid organs (spleen, thymus, Peyer’s Patches and lymph nodes) [12].

**Table 1:** Brief history of cancer and the lymphatic system

Time period	Significance
1700’s to mid-1800’s	Lymph theory: Cancer arose in tissue “lymph” and metastasized to palpable lymph glands
1850’s	Cellular theory: Cancer originates in abnormal cells – Virchow “When the axillary gland becomes cancerous after disease of the mamma… during a long period remains diseased without the glands next in succession or other organs becoming affected, the gland collects the hurtful ingredients absorbed from the breast and affords protection to the body… but at length proves insufficient, perhaps itself becomes a new independent source of further propagation of the poisonous matter from the diseased part of the gland.”- Rudolf Virchow [10] Lymphogenous metastasis to contiguous lymph nodes – Subsequent radical removal of primary cancer and regional lymph nodes
1960’s	Cancer as systemic disease – emphasis turned to hematogenous metastasis– chemotherapy added.
1900’s	Sentinel node concept – less extensive lymphadenectomy
2000’s	Immune system influence on cancer leading to immunotherapy and resurgence of interest in cancer microenvironment on growth, dormancy and metastasis
2018	Nobel Prize in Physiology or Medicine to James P. Allison and Tasuku Honjo for discovery of CTLA- 4 and PD-1 respectively
2022	“Lymphatic systemomics and cancer” – lymphatic vessels, lymph circulation, immune system cells/nodes, route of entry and transport – overseer/influencer of entry/exit and barometer of cancer microenvironment (physiochemical state of ECM, resident and trafficking cells, extracellular ‘molecules’)
2023	Nobel Prize in Physiology or Medicine to Katalin Kariko and Drew Weissman for the development of mRNA vaccines and the immune system

Lymphology with imaging of lymphatic system anatomy and function, has shed light to link cancer and metastasis [13, 14]. Also, lymphatic endothelial biology became a subject of considerable interest [15]; Lymphangiogenesis [16–18] along with the intensive attention to hemangiogenesis as key to cancer growth was shown to be perhaps a more important contributor to cancer growth and metastasis. Still, the molecular players were not yet known [19, 20].

The next breakthrough was the SLN concept - that the cancer cells metastasize to specific lymph nodes in the chain and inspection of these alone could predict whether metastasis would occur. For more detailed discussion, see following sections.

In the past two decades with the advancement of tools from the Human Genome Project, an array of genes and proteins have been discovered that influence the growth and development of the lymphatic system [20]. The signaling pathways uncovered overlap with those known to be involved in cancer and in benign tumors/lymphatic malformations [19]. These now provide the molecular lymphology insight that help to explain the “lymphangiogenesis and lymphogenic syndromes” scheme proposed nearly forty years ago [9].

The past two decades have also seen a rethinking of the cancer cell in the cancer microenvironment of host cells of various types (next section), their products – e.g. cytokines, glycoproteins, exosomes, and extracellular matrix, particularly hyaluronan. Changes in this microenvironment influence whether the cells will remain latent or migrate and proliferate through epithelial-endothelial mesenchymal transition (EMT). On a molecular level, as Jackson et al. have demonstrated [21, 22], the LYVE-1 hyaluronan receptor on lymphatic endothelium is the key entry point for trafficking immune cells as well as hyaluronan coated-cancer cells. In this way, the initial lymphatic capillary is not only a barometer of the cancer microenvironment but also governs whether the cancer cells will be able to metastasize.

Less than a decade ago, but extending back more than a generation, the immune cell population surrounding the cancer has been recognized as crucial to the events that follow - leading to immunotherapy and a dramatic breakthrough in the therapy of certain cancers such as melanoma.

**Fig. 1:**
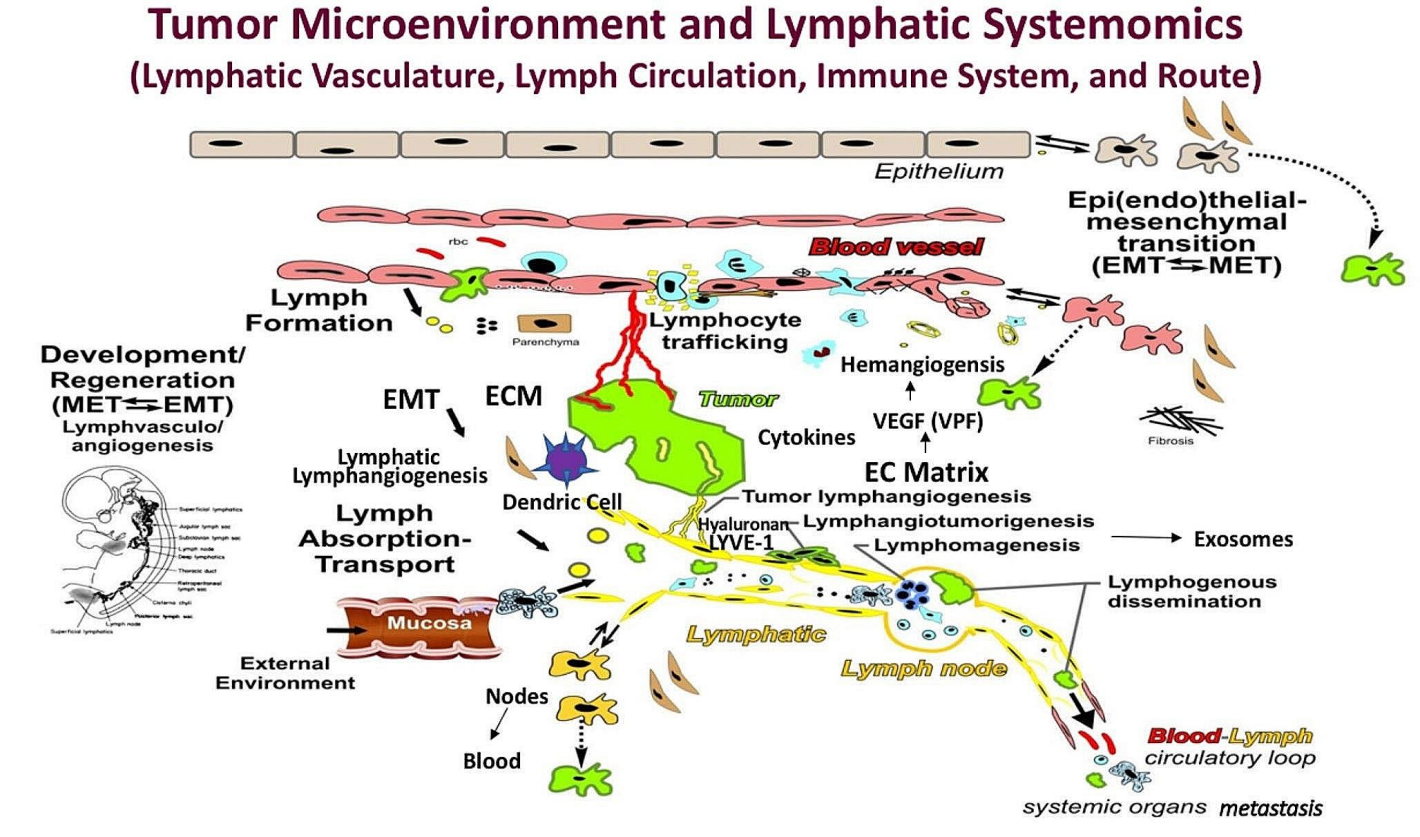
Tumor Microenvironment and lymphatic sytemomics. Points of interplay between the developing cancer and ongoing processes within the interstitium and lymphatic system; lymphedema, lymphangiogensis, tumor-generated immune response. Potential sites of epi/endothelial-mesenchymal transition (EMT) and the reverse process (MET) in development/regeneration and neoplasia (green) are identified. These complex structural-functional interactions participate in the pathogenesis, clinical manifestations, evaluation, and prognosis as well as the treatment of cancer. Permission has been obtained from the following article: Witte, M et al. (2012) Clin Exp Mets 29: 707–712

Thus, in summary, lymphatic systemomics has intersected with cancer biology to integrate the various theories of cancer and its relation to the lymphatic system. These multifaceted and complex relationships [23] - from blood capillary hyperpermeability (VPF = VEGF) [24] to cell populations in the microenvironment, associated EMT events, matrix changes, interstitial cell populations, and cytokines all influence conditions for cancer cell entry into lymphatics to launch the process of metastasis (Fig. 1).

Virchow, nearly 170 years ago, before any molecular understanding, envisioned these connections and contemplated the myriad of events that surround the cancer cell. Whether the regional lymph nodes would welcome or restrain the cancer cell and act as a locus for “harmful ingredients” and “poisonous matter” transferred from the primary cancer promoting metastasis to distant sites had been entertained [10].

### The association between cancer metastasis and hemangiogenesis versus lymphangiogenesis

**Stanley P. Leong**

The relationship between cancer cells and the vascular systems, including both lymphatic and blood vessels, plays a pivotal role in cancer metastasis. This process is governed by a sophisticated network of genetic, molecular, and cellular elements. Genetic alterations in cancer cells can amplify their capacity to invade and migrate through blood and lymphatic channels. Such mutations might activate genes that drive cancer growth (oncogenes) or disable genes that suppress it, leading to the increased production of substances that encourage the formation of new blood and lymphatic pathways, facilitating cancer metastasis [25].

Changes at the genetic level can also cause cancer cells to produce more of certain molecules on their surface that help them stick to the inner walls of blood and lymphatic vessels. Molecules like selectins, integrins, and those belonging to the immunoglobulin superfamily are critical for the early stages of metastasis [26].

Cancer cells release enzymes such as matrix metalloproteinases (MMPs) that break down the surrounding extracellular matrix, making it easier for them to invade nearby tissues and enter into the bloodstream or lymphatic system [27]. Cancer and stromal cells within the cancer microenvironment produce signaling molecules like chemokines and cytokines, which can foster cancer expansion, inflammation, and the creation of areas in distant tissues that are receptive to cancer cells. These molecules also guide cancer cells as they move through and out of blood and lymphatic vessels [28].

**Fig. 2:**
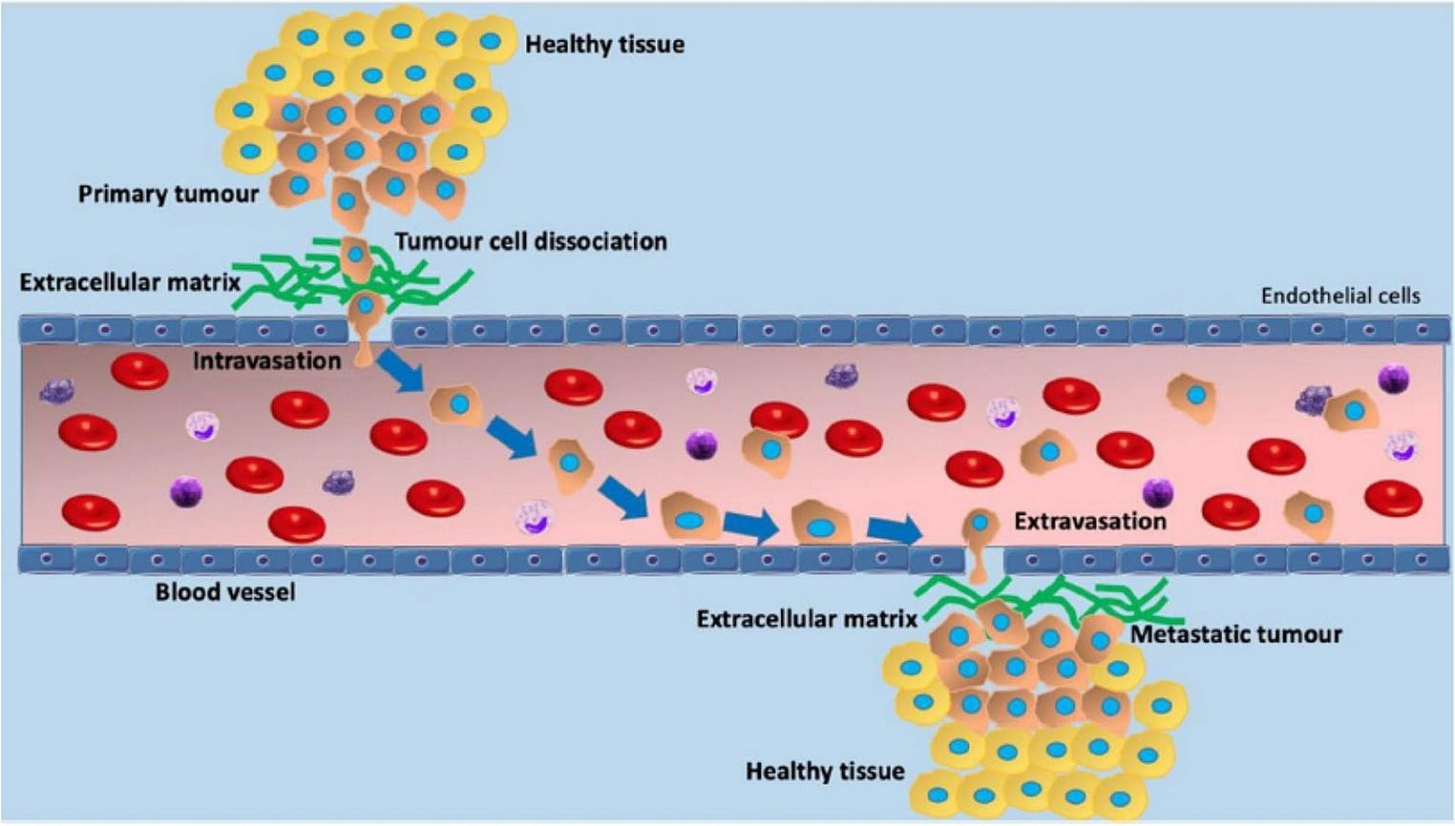
The metastatic process involves several critical steps where cancer cells leave the primary site, breach the nearby tissue, and gain entry into adjacent blood or lymphatic vessels—a phase known as intravasation. In this figure, a blood vessel is depicted. Once these cancer cells infiltrate the vascular system, their ability to halt and cling to the inner lining of the blood vessels becomes crucial, setting the stage for their subsequent exit from the bloodstream. A portion of these cells successfully bind to the walls of blood vessels and manage to move out of the bloodstream and into the surrounding tissue. In this new location, they have the potential to initiate secondary metastatic growths. For circulating cancer cells to transition in and out of the bloodstream and navigate through it, they must attach themselves to the inner surface of the blood vessel and maneuver through the endothelial wall cells. Reproduced with permission. Vasilaki D, Bakopoulou A, Tsouknidas A, Johnstone E, Michalakis K (2021) Biophysical interactions between components of the tumor microenvironment promote metastasis. Biophys Rev 13 (3):339–357. 10.1007/s12551-021-00811-y

Cancer cells emit substances like the vascular endothelial growth factor (VEGF) to spur new blood and lymphatic vessel growth, offering a pathway for cancer cells to metastasize. Variants of VEGF, such as VEGF-A and VEGF-C/D, are implicated in blood vessel and lymphatic vessel growth, respectively [29]. Cancer cells enter small vessels with a thin wall consisting of one cell thick of endothelial cells. The entry of cancer cells into vessel channels involves complex interactions with the vessel-lining endothelial cells and other types of cells, like pericytes and immune cells. Cancer cells may employ various strategies to penetrate the vessel barrier including invasion and intravasation into the blood vessel [30]. To complete the metastatic cascade, cancer cells within the blood vessel may undergo extravasation through the endothelial cells of the blood vessel to invade the surrounding normal tissue and establish a metastatic focus as shown in Fig. 2.

**Fig. 3:**
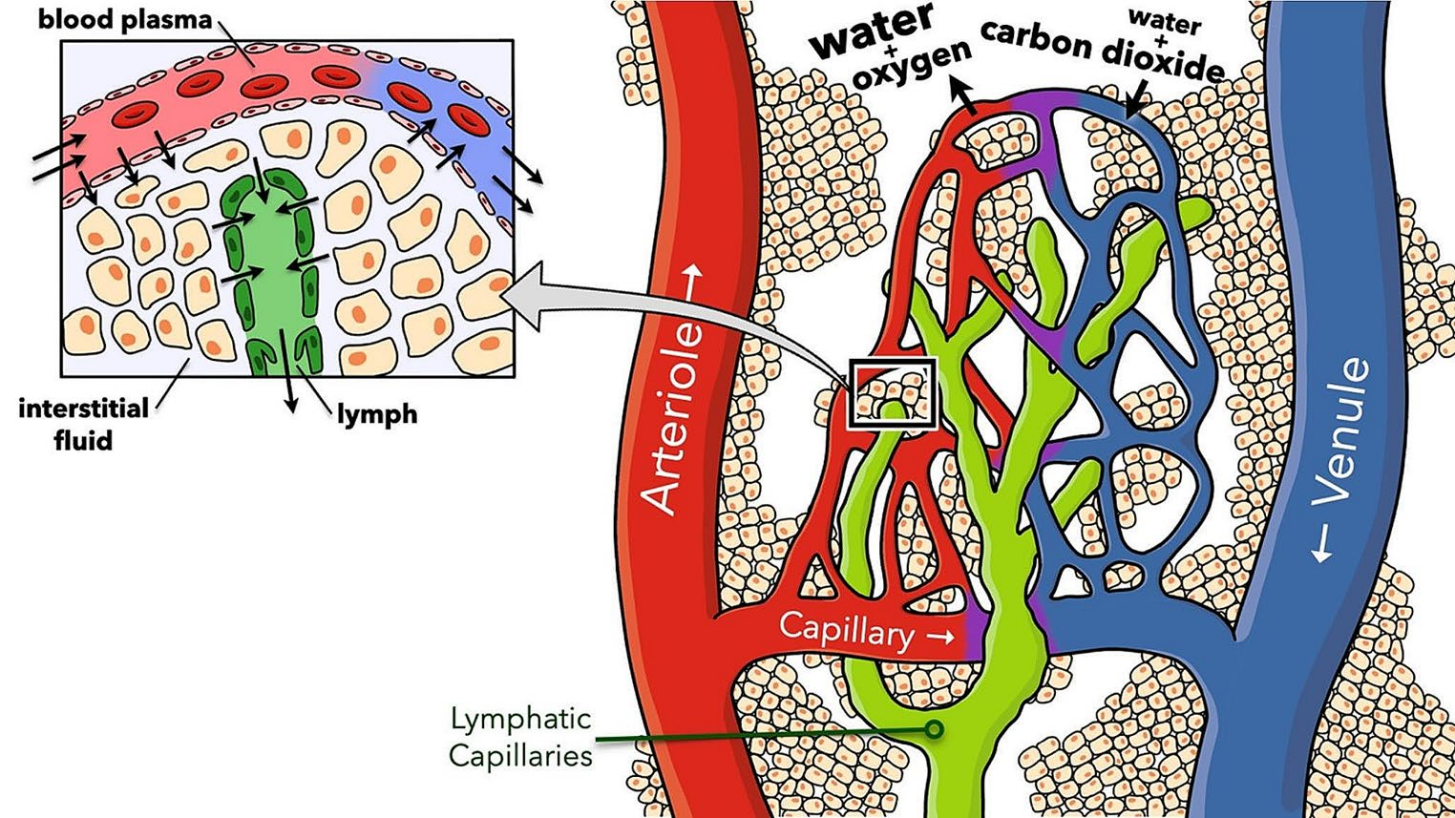
The capillary junction shows the connection between the arteriole and venule. The diameter of the capillary is about 8–10 microns. The capillary vessel consists of a single layer of flattened endothelial cells. The diameter of the post-capillary venule is about 30 micrometers, large enough for cancer cells with an average diameter up to 20 micrometers to squeeze through the post-capillary venule. The lymphatic capillary is slightly larger with a diameter varying from 10 to 80 microns. It consists of a single layer of lymphatic endothelial cells with valves to allow the flow of lymph in one direction. See Fig. 4. Reprinted with permission from Justin Seibert of Seibert Science. Science S (2024) Lymphatic System. https://youtu.be/X2hHK1BHV2E?si=ywPqXIZorj3sZj_u

**Fig. 4:**
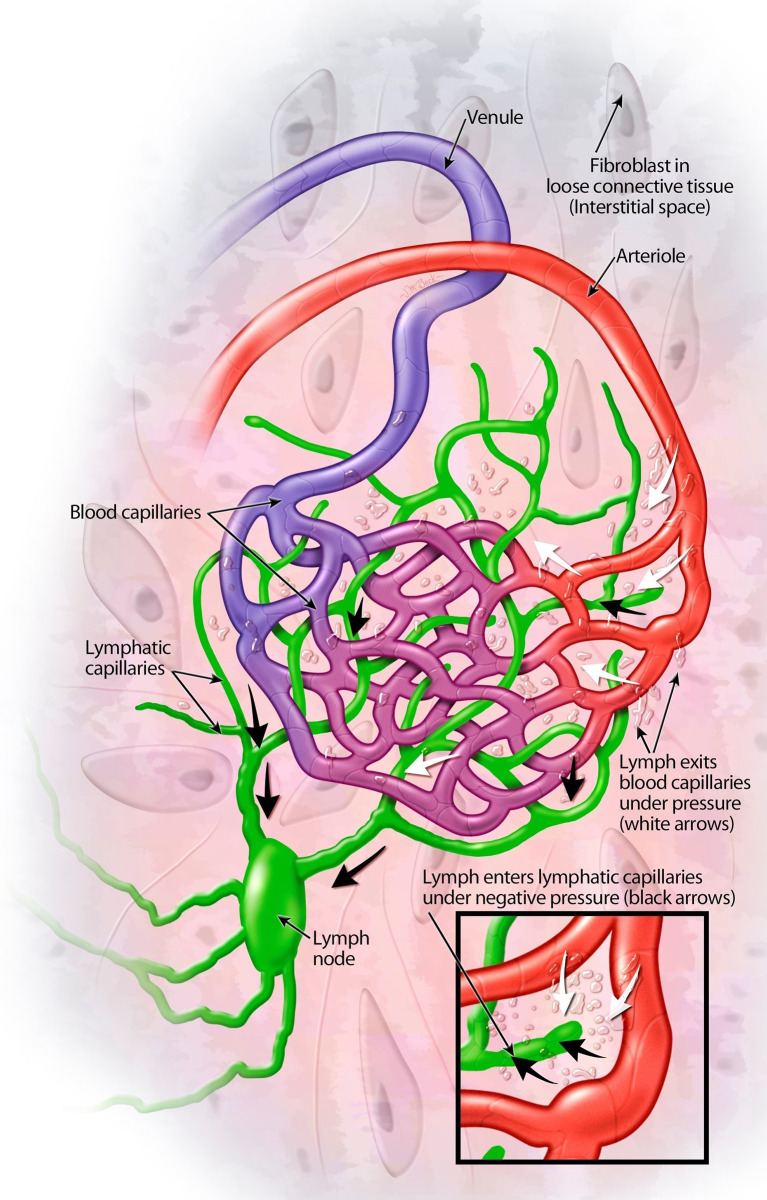
Movement of lymph from blood to lymphatic capillaries with valves to alllow lymph to flow only in one direction. Lymph leaves blood capillaries under osmotic pressure (white arrows). In the right lower diagram, lymph enters lymphatic capillaries under negative pressure (black arrows) and travels to the lymph nodes via the afferent lymphatic vessels and exits through the efferent lymphatic vessels. Reprinted with permission from Tactile Medical. On a daily basis, 17 liters of the 20 liters of blood at the arterio-venous capillary junctions return through the venous capillary circulatory system. About 3 liters of fluid, without the cellular components of blood, escape into the extracellular space and drain into the lymphatic vessels. This fluid, known as lymph, carries cellular debris, protein macromolecules, excess water, and toxins. Lymphedema occurs when the extracellular fluid is not adequately drained. The lymph fluid is filtered through multiple lymph nodes (about 600 in a normal person) before it finally drains into the thoracic duct on the left neck and into the subclavian vein and into the jugular vein on the right neck where it re-enters the vascular system as sterile lymph fluid (https://www.youtube.com/watch?v=I7orwMgTQ5I)

Hemangiogenesis results in the formation of new blood vessels for cancer growth and spread [31]. For the cancer cells to enter the blood circulation, the cells must enter the venous circulation through the smallest venules with one cell wall at the junction of the arteriovenular capillaries (Fig. 3 and 4), pass through the heart and lungs and into the systemic arterial circulation to systemic sites [32]. Alternatively, cancer cells can migrate through the lymphatic system to the thoracic duct in the left neck and lymphatic channel in the right neck, then, enter the venous system via the subclavian veins. Once inside the vessels, cancer cells need to withstand the flow’s mechanical forces and avoid being detected and destroyed by the immune system. They often clump together with platelets and other blood cells to improve their chances of survival. The exit of cancer cells from the bloodstream or lymphatic system to establish new sites mirrors their entry process but in the opposite direction. They must attach to and then move through the vessel wall into the new tissue [33].

Solid cancers frequently first metastasize to nearby lymph nodes via the lymphatic system. Cancer cells can establish residence in lymph nodes, multiply to form new growths, or move on to other parts of the body through lymphatic channels or the bloodstream [34, 35].

The process of metastasis, as described above, is evident in several types of cancer, each demonstrating unique pathways and mechanisms for dissemination as described below:

Breast cancer commonly metastasizes first to nearby lymph nodes before reaching distant organs like the bones, liver, lungs, and brain. The cancer cells use molecules like E-selectin and integrins to attach themselves to the inner walls of vessels. Growth factors such as VEGF-C and VEGF-D are key in stimulating the growth of new lymphatic channels, facilitating the journey of cancer cells to the lymph nodes [36].

Melanoma, known for its propensity to rapidly metastasize, aggressively invades both lymphatic and blood vessels. It produces high levels of enzymes (like MMPs) that break down tissue barriers [37], and growth factors (including VEGF-A and VEGF-C) that drive the growth of new vessels, aiding in the metastasis of cancer cells. Melanoma progression has been correlated with hemangiogenesis [38]. Although several studies have shown that cancer density of the microvessels has been correlated with decreased disease-free and overall survival [39], other studies showed no differences of cancer microvessel density in primary or metastatic melanomas [40]. Thus, the predictive value of cancer hemangiogenesis in melanoma remains controversial [41]. Currently, the potential prognostic utility of hemangiogenesis in melanoma is not clear [42].

Colorectal cancer (CRC) CRC tends to metastasize to the liver via the portal vein and to the lungs through systemic circulation. The cancer cells express various molecules that enable them to stick to and move through vessel walls, with the CXCL12/CXCR4 pathway playing a significant role in directing their migration to specific sites [43].

Lung cancer (NSCLC) is notorious for metastasis to various organs, including the brain, bones, liver, and adrenal glands. The formation of new blood vessels, a process driven by VEGF, is crucial for the progression and metastasis of lung cancer. These cancer cells also have a high expression of molecules that facilitate their movement into distant tissues [44].

Prostate cancer often finds its way to the bones via the bloodstream, secreting factors like VEGF and TGF-β that not only promote the formation of new blood vessels but also remodel bone tissue, creating a conducive environment for metastasis. The interplay between prostate cancer cells and the bone environment involves a complex network of molecules and pathways that regulate bone formation and resorption [45].

Ovarian cancer primarily metastasizes within the peritoneal cavity but can also move to distant sites through lymphatic and blood vessels. The shedding of cancer cells into the ascitic fluid, expression of molecules like CA125 and integrins, and secretion of MMPs and VEGF are key mechanisms that facilitate its invasion and metastasis [46].

Head and neck squamous cell carcinoma (HNSCC) HNSCC often extends to regional lymph nodes via the lymphatic system. The cancer cells express molecules that help them attach to and break through the extracellular matrix and vessel walls, with VEGF-C playing a significant role in stimulating the growth of new lymphatic vessels [47].

These examples underscore the shared, yet distinct strategies employed by different cancers to navigate the lymphatic versus blood systems and establish new metastatic sites. In the context of the primary cancer site, cancer cells generally favor the lymphatic system over blood vessels relating to the initial process of metastasis. This preference is attributable to several reasons as listed below:

Reduced flow resistance the lymphatic system’s flow is gentler with less resistance compared to the vigorous flow in blood vessels, facilitating easier entry and survival of cancer cells within lymph channels.

Cancer cells are more likely to stimulate the growth of new lymphatic channels around them through the production of factors like VEGF-C and VEGF-D, which specifically encourage lymph vessel development.

Lymphatic vessels have comparatively thinner walls and more loosely connected cells than blood vessels, lacking a cohesive basement membrane, which eases the penetration of cancer cells.

Many tissues supporting common cancers are already equipped with extensive lymphatic vessels, offering a path of least resistance for cancer cell migration.

Cancer cells may engage with and potentially manipulate the immune system to facilitate the process of metastasis.

Cancer cells through the lymphatics often culminates in the lymph nodes, which can act as initial sites for cancer cell accumulation and subsequent metastasis. The involvement of lymph nodes is a critical factor in assessing cancer progression.

While the lymphatic route maybe typically the initial pathway for cancer metastasis, it’s crucial to recognize that the bloodstream is the conduit for the distant organ invasion, marking a more severe stage of cancer metastasis. The preference for either the lymphatic or blood route can vary on the cancer type as mentioned above, its origin, and specific genetic traits that may enhance its adaptability to the bloodstream.

**Fig. 5:**
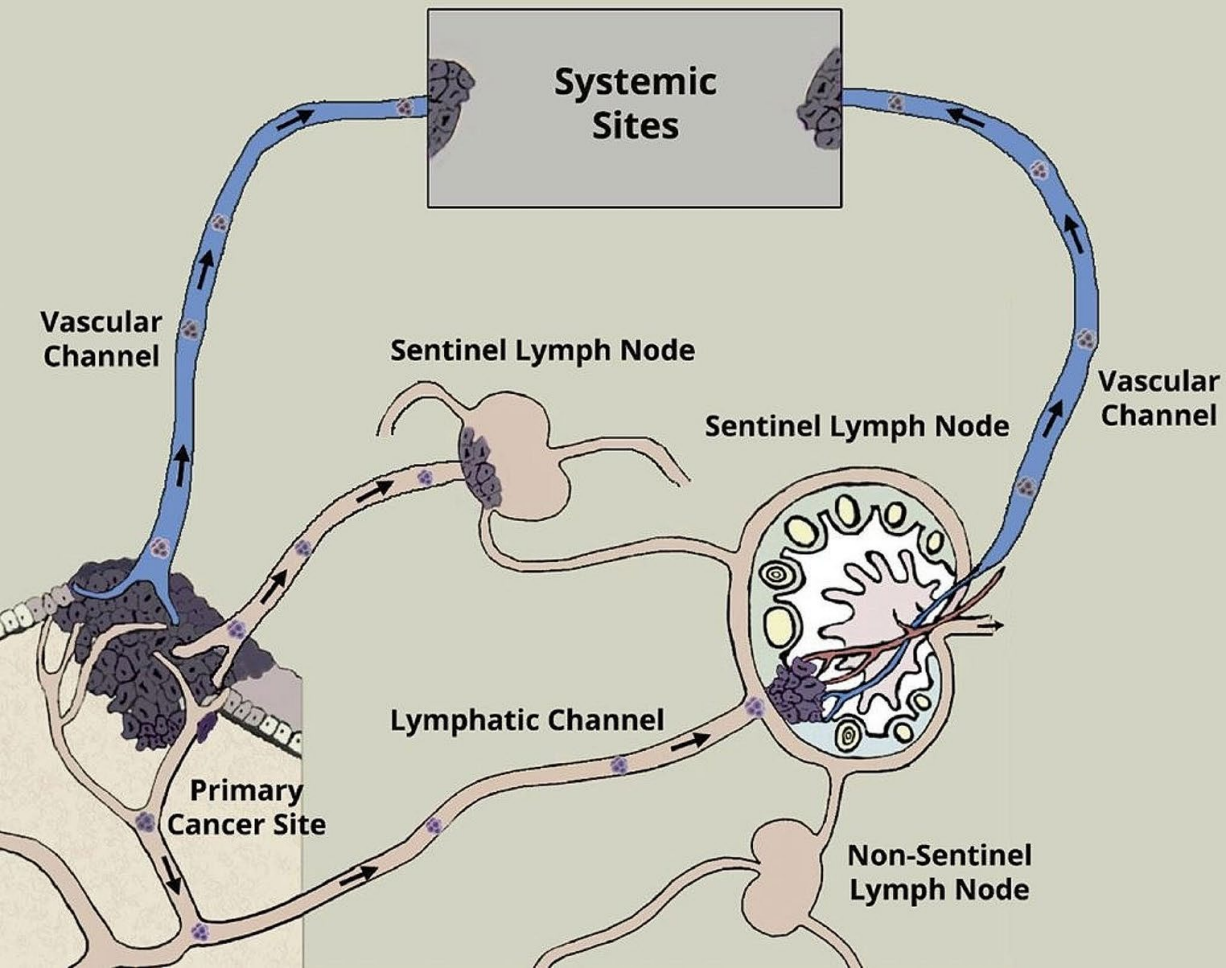
Dichotomy of routes of cancer metastasis: one through the lymphatic vessels to the sentinel lymph nodes as the primary gateway and the other through the blood vessels directly to the distant sites. Permission has been obtained to reproduce this figure from Springer Nature from the cover image for the Special Issue of Clinical and Experimental Metastasis, Springer Nature, Volume 35, Number 5–6, 2018

**Fig. 6:**
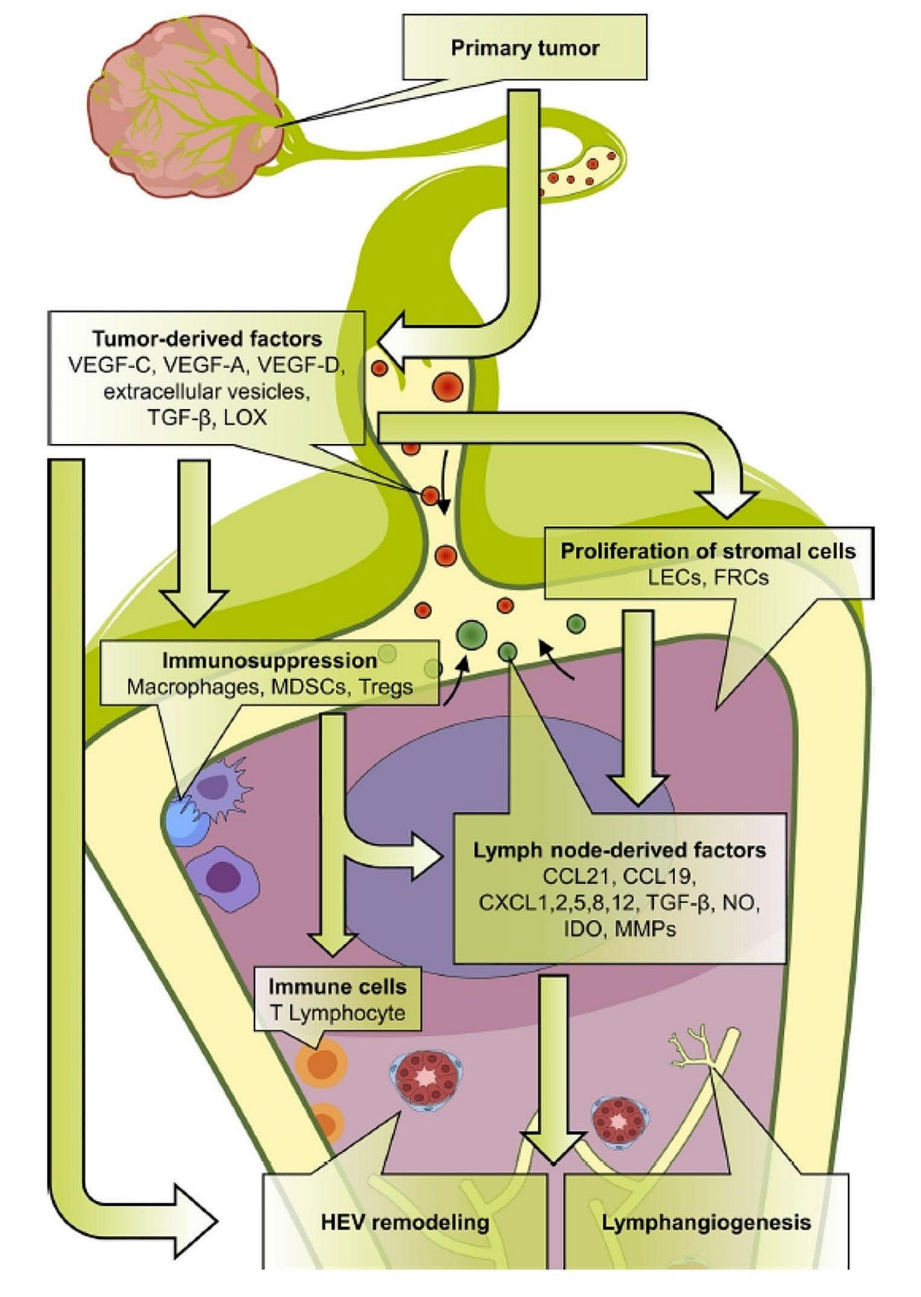
Establishment of the lymph node (LN) pre-metastatic niche. Tumor-derived factors, including vascular endothelial growth factor (VEGF-A, VEGF-C and VEGF-D), extracellular vesicles, TGF-β and lysyl oxidase (LOX), induce an immunosuppressive microenvironment by recruiting macrophages, myeloid-derived suppressor cells (MDSCs) and regulatory T cells (Tregs). Proliferation of lymphatic endothelial cells (LECs) and fibroblastic reticular cells (FRCs) drives the production of LN factors such as chemokines (CCL19; CCL21; CXCL1, 2, 5, 8, and 12); TGF-β; matrix metalloproteinases (MMPs); indoleamine-2,3-dioxygenase (IDO); and nitric oxide (NO), which induce high endothelial venule (HEV) remodeling, stimulate lymphangiogenesis, and regulate tumor cells chemoattraction at metastatic stage. Permission to use this figure from Cellular and Molecular Life Sciences, Gillot et al., 2021, falls under Creative Commons CC BY 4.0. Gillot L, Baudin L, Rouaud L, Kridelka F, Noel A (2021) The pre-metastatic niche in lymph nodes: formation and characteristics. Cell Mol Life Sci 78 (16):5987–6002. 10.1007/s00018-021-03873-z

A deep understanding of these intricate interactions is crucial for devising treatments that can interrupt the metastatic process, such as drugs that block the enzymes cancer cells use to invade tissues, inhibit the growth of new blood vessels, or target the specific molecules and pathways cancer cells use to metastasize. These complicated pathways of cancer metastasis [7] may be depicted in Figs. 5 and 6.

## The role of sentinel lymph node in cancer metastasis

**Stanley P. Leong**

The development of SLN biopsy in penile cancer by Cabanas [48], in melanoma by Morton [49] and in breast cancer by Giuliano [50] has revolutionized the treatment of cancer relating to the resection of regional lymph nodes. The radical approach to remove all the draining lymph nodes of Halsted [11] to simply sample the SLNs has resulted in about 80 to 85% of the melanoma patients [51] avoiding radical lymph node dissection. Based on the MSLT-II study, melanoma patients with a small cancer burden of 0.6 mm or less in the SLN would be spared of a completion lymph node dissection [51]. In breast cancer, with clinically negative lymph nodes, the positive SLN biopsy rate is about 20% [52]. However, axillary lymph node dissection has been avoided in most of the cases based on the randomized study that there is no survival difference between the SLN versus axillary lymph node dissection group [53]. Thus, these change of practices has significantly reduced the incidence of lymphedema in melanoma [54] and breast cancer [55].

In the pre-sentinel node era, according to Cady [56], prophylactic resection of regional lymph nodes with or without metastases showed no improved cure rates relative to the observation group. Thus, Cady concluded that the lymph node metastasis was the marker but not the governor of cancer survival. The disadvantage of this analysis is that these patients were heterogeneous with macrometastatic disease. From the SLN studies, the patient groups are more uniform, and the SLN is more specific within the regional lymph node basin as the most likely lymph node receiving the cancer cells from the primary site. Furthermore, the cancer burden in the SLN is microscopic and patients with a negative SLN biopsy represents those whose cancer has not metastasized to the lymph node in most cases.

### What do we learn from patients with a negative sentinel lymph node biopsy?

**Stanley P. Leong**

Both melanoma and breast cancer metastasize through the lymphatic vessels in a more orderly fashion [57] than cancers of the internal organs such as the stomach, pancreas, lungs, and other organs, which show more complicated lymphatic drainage pathways [58]. Both melanoma [59, 60] and breast cancer patients [61] have a worse disease-free and overall survival with a positive SLN biopsy. The unique advantage from the SLN biopsy is that it allows us to understand the biology of early cancer reaching the SLNs with minimal cancer burden. Patients with a negative SLN biopsy represent that, in most instances, their cancer cells have not yet metastasized to the SLNs. A low recurrence rate of 5–10% [62, 63] is associated with melanoma patients with a negative SLN biopsy. The recurrence rate is also very low, less than 5% [61] for breast cancer patients with a negative SLN biopsy. In another study by Quiet et al. [64], the authors concluded that with extended follow-up evaluation, node-negative breast cancer is a curable disease. For colon cancer, with a negative SLN biopsy, most patients may be cured without systemic treatment [65]. For upper GI cancers, lesser surgery may be performed with a negative SLN biopsy [66].

In the 8th Edition Cancer Staging Manuel [67], it has been shown that Stage IIIA melanoma patients tend to do better than Stage IIB and IIC relating to survival, suggesting that certain primary features of melanoma such as Breslow thickness, ulceration, mitotic rate and microsatellitosis may increase the chance of melanoma metastasis to systemic sites even though the SLN biopsy is negative. These high-risk characteristics may contribute to metastasis through the blood vessels.

Further, the false negative rate of melanoma SLN biopsy ranges between 6-21%. Thus, some patients with a negative SLN biopsy may have possibly a positive SLN biopsy.

In a recent phase 3, double-blinded, randomized and placebo-controlled Lancet study, Keynote-716 [68], the effect of adjuvant therapy by pembrolizumab (anti-PD-1) in melanoma patients with Stage IIA and IIB (TNM stage T3b or T4 with a negative SLN biopsy) has been evaluated. It has been shown that Pembrolizumab as adjuvant therapy for up to approximately 1 year for stage IIB or IIC melanoma resulted in a significant reduction in the risk of disease recurrence or death versus placebo, with a manageable safety profile. However, only about 20% of the patients were evaluated. In addition, the study was not able to address the subgroups of patients with regional versus systemic metastasis as well as from different sites of the primary melanoma. Based on the Keynote-716 study, it appears that a minority of melanoma patients with Stage IIB and IIC with a negative SLN biopsy may develop systemic metastasis, thus, it is important to understand the characteristics of this group of patients and the metastatic pathways either through the lymphatic vessels to the SLN(s) or through the blood vessels to the systemic sites.

According to Fisher [69], breast cancer is a systemic disease. Lymph node involvement is not orderly contiguous extension, but rather a marker of distant disease. Systemic metastases are multiple and widespread. Under these circumstances, treatment of local or regional disease should not affect survival. However, in the SLN era, patients with a negative SLN biopsy fare much better than those with a positive one suggesting that, perhaps, the SLN serves as a gateway in many of the cases. Cancer in its early stage without the involvement of SLN may be localized and can be potentially cured by surgical resection. Thus, cancer development is progressive according to the spectrum theory of Hellman [70]. Not only is there a spectrum of malignancy, but there is an accompanying spectrum of potential curative treatments. Cancers early in their progression should be amenable to localized therapy. Patients with oligometastases, either de novo or following systemic treatment, should be cured by ablation of these lesions. This paradigm emphasizes the importance of specific characteristics related to where in the spectrum of malignancy an individual cancer is compartmentalized. Truly localized and oligometastatic versus wildely metastatic cancers are likely to require different treatment strategies. Surgery or radiation therapy may result in curative treatment of such oligometastases either alone or combined with systemic therapy. Therefore, the patterns of metastasis from the SLN point of view, spectrum theory seems to be more compatible with the SLN being the major gateway in most of the cases, as most of patients with a negative SLN biopsy tend to do well without the development of metastatic disease.

### The sentinel lymph node may be a major gateway to cancer metastasis

**Stanley P. Leong**

In mouse models from recent studies, cancer cells were shown to invade blood vessels within the SLN, enter the blood circulation and establish metastases in the systemic sites [71, 72]. It seems likely that there are various patterns of metastasis from the primary cancer site to the systemic circulation (Fig. 5). Some cancers may metastasize from the primary site to the distant sites [73] while others can only enter the systemic circulation through the SLNs. As mentioned above, for a variety of cancers including melanoma and breast cancer, the incidence of metastasis to the distant sites in patients with a negative SLN biopsy is quiet low suggesting that SLN may play an important role in systemic metastasis. In melanoma [35, 58] and breast cancer [74–76], the dominant method of metastasis seems to be through the SLN, which may serve as a major gateway for systemic metastasis. Future studies will need to address the molecular mechanisms of cancer leaving the primary site to the SLN and then to the distant sites. Are different clones involved during each stage of metastasis? For those cancer cells preferring metastasis through the blood vessels into the systemic circulation using the VEGF-A and VEFGR-2 axis, are they different from the SLN-bound clones using VEGF-C and VEGFR-3 axis [77] with different genetic and molecular profiles? Perhaps, spatial imaging and single cell analysis may be able to unlock the differences among these cancer clones [78, 79]. These molecules may potentially be targeted for therapeutic benefits to control or stop cancer or even reverse metastasis [80]. In fact, using single-cell RNA sequencing to analyze the comprehensive transcriptome of lymphatic endothelial cells (LECs) in murine skin draining lymph nodes. Fujimoto et al. have found new markers and functions of distinct LEC subpopulations [81]. These LECs in the subcapsular sinus of the lymph node have been found to be associated with rapid lymphocyte egress from lymph nodes. Recently, it has been demonstrated that LECs may respond and affect the immune response [82]. Overall, the lymphatic system is a complex network of lymph flowing through the lymph nodes and the lymphatic system may serve as a conduit for cancer metastasis [58]. The thoracic duct as mentioned above is vital to the body’s lymphatic system. It will not be surprising that cancer cells may travel through the thoracic duct into the blood circulation.

Understanding the molecular events leading to the formation of a pre-metastatic niche [Fig. 6] in SLNs may explain the conundrum of a SLN being reactive against cancer and as an incubator for cancer growth. A more detailed account of the pre-metastatic niche may be found in several reviews [76, 83, 84]. Once these molecules are targeted, therapies may be developed to disrupt the cancer metastasis process and potentially prevent cancer metastasis to distant organs.

### Lymphangiogenesis in sentinel or regional lymph nodes

**Stanley P. Leong**

Based on experimental and clinical studies, cancer lymphangiogenesis has been found to be significantly correlated with poor prognosis [85]. Two major recent advancements have opened new inroads in the understanding of cancer lymphangiogenesis and cancer metastasis. These include clinical significance of SLNs in melanoma [86] and breast cancer [50] and the discovery of lymphatic markers such as VEGF-C, LYVE-1, podoplanin, and Prox-1 [18]. The lymphatic system may be considered the major conduit for cancer metastasis [58].

Circulating lymphocytes travel between the blood and structures such as lymph nodes, Peyer’s patches and spleen, where antigens and antigen-presenting dendritic cells are present. When the lymphocytes migrate through the lymph nodes, they bind to the high endothelial venules (HEVs) with cuboidal endothelial cells and enter the circulation. The HEVs are uniquely different from the normal venules in 2 ways: (1) HEVs express unique adhesion molecules or vascular addressins, acting as ligands for homing receptors of the lymphocytes; (2) chemokines and chemokine-binding molecules are generated within the extracellular matrix near the HEVs. These chemokines induce production of integrins on circulating lymphocytes and draw them into the lymph nodes and Peyer’s patches to initiate effective immune responses with aid from the antigen-presenting dendritic cells [87]. Lymphocytes may leave the efferent lymphatic channels to a downstream lymph node and then gain access to the circulation using the pathways through the HEVs as mentioned above [88]. A detailed account of the molecular mechanisms of lymphoctye trafficking and migration through the HEV is discussed in a separate review [76]. Jackson has described a newly emerging mechanism for lymphatic entry into the lymph node involving the large polysaccharide hyaluronan and its key lymphatic and immune cell receptors LYVE-1 (Lymphatic Vessel Endothelial receptor) and CD44. This mechanism may also be used by hyaluronan cancer cells to metastasize to the lymph nodes [89].

VEGF A, B, C and E bind with their respective receptors and cause proliferation of blood vessels while VEGF C and D are involved in lymphangiogenesis [90] as shown in Fig. 6. Based on their extensive studies of lymphangiogenesis and cancer metastasis, Detmar and Hirakawa have concluded that VEGF-C and the VEGFR-3 axis play an important role in the lymphangiogenesis of cancer metastasis to allow cancer cell to enter through the lymphatic vessels [77]. Cancer-induced lymphatic vessels by lymphangiogenesis may become dilated [91]. In the lymphatic vessel cancer cells may be trapped at the valve with subsequent growth within the lymphatic vessel [92]. Cancer cells may extravate through the wall of the lymphatic vessel and invade into the adjacent soft tissue (Fig. 2).

Cancer lymphangiogenesis may be considered as a marker for cancer metastasis. In the cancer microenvironment, VEGF C/D being produced by cancer cells promote local lymphangiogenesis associated with the formation of new lymphatic vessels. SNAIL1/2 downregulates E-cadherin and promotes epithelial to mesenchymal transition in cancer cells resulting in enhanced invasiveness of cancer cells. Cancer cells may enhance their invasiveness through epithelial–mesenchymal transition [93, 94]. TWIST1 plays an important role in metastasis enhancing invadopodia and extravasation. Further, cancer cells can upregulate the production of CCR7/8 and CXCR4/5, which interact to the corresponding receptors from the lymph node namely CCL1/21 and CXCL10/12 respectively, resulting in the migration of cancer cells to the lymphatic vessels by chemotaxis. ALOX15 is produced by some cancer cells, acting as a catalyst to convert arachidonic acid to 12[S]-HETE and 15[S]-HETE, which cause circular defects on LECs to let cancer cells enter the lymphatic vessels. By mechanical means, flow of the interstitial fluid forces the cancer cells into the lymphatic vessels. Further, at the edge of the cancer microenvironment, interstitial flow and lymphatic drainage are increased [95, 96]. In addition, the interstitial flow induces cancer cells to produce and respond to the autocrine chemokine gradients towards the lymphatic vessels [97].

Stromal cells may also be affected by interstitial flow with changes of the alignment in cell and matrix [76, 97], increase motility of fibroblast by matrix metalloproteinase-1 [98], and myofibroblast differentiation to become cancer-associated fibroblast through the action of transforming growth factor (TGF)-b1 [99]. Cancer-associated fibroblast and collagen degradation enhance cancer invasion into the lymphatic vessels [100]. Swartz and Lund have asserted that cancer invasion within the cancer microenvironment is a multifactorial process including lymphangiogenesis, interstitial flow mechanics, and immune responses [101]. Lymphangiogenesis may not just be present in primary cancer site but also may occur in other metastatic sites [102]. Thus, lymphangiogenesis and the remodeling of lymphatic vessels play an important role in cancer metastasis [103], thus, facilitating the entry of cancer cells into the lymphatic vessel. It has been acknowledged while lymphangiogenesis is associated with cancer invasion and poor prognosis, checkpoint inhibition immunotherapy may be employed for therapeutic benefit [101].

Although the clinical significance of SLNs in melanoma [104] and breast cancer [50] has been well established, advancement in molecular biology and recent development of the lymphatic and blood vessel biomarkers may allow us to track cancer metastasis from the primary site to SLNs on a molecular level. The challenge is to further define these molecules in a more detailed format so that therapeutic modalities may be developed to block these molecules.

The role of SLN in causing distant metastases in patients requires further investigation with an effort to identify the molecules relevant to underlying mechanisms of metastasis with the goal to block them for therapeutic benefit.

### Spread of sarcoma; why primarily via the blood vessels rather than the lymphatic vessels

**Stanley P. Leong**

Soft tissue sarcomas are rare cancers derived from mesenchymal origin. Most sarcoma subtypes do *not* metastasize to the lymph nodes, in distinct contrast to lymph node metastasis as a major route in melanoma and carcinoma. Exception to this general rule, several subtypes of sarcoma can develop lymphatic metastasis, and these include rhabdomyosarcoma, synovial sarcoma, epithelioid sarcoma, clear cell sarcoma, and angiosarcoma [105–108]. On the other hand, hematogenous metastasis to the lung is more frequent for most subtypes of sarcoma with the lung being like a filter from the venous circulation. The mesenchymal origin of sarcoma may be associated with its inclination for hematogenous rather than lymphatic metastasis like melanoma and carcinoma. Although SLN biopsy has been proposed as assessing the lymph node basin in patients with high-risk sarcoma histology [109], because of the relatively rare occurrence of these subtypes, SLN biopsy is not often applicable to sarcoma. The molecular mechanisms why sarcoma is more prone to hematogenous rather than lymphatic metastasis as compared to melanoma and carcinoma are not well understood. The anatomic relationship between sarcomas and the major lymphatics and blood vessels needs to be studied to assess potentially the role of these adjacent vessels may play a role in the metastasis of sarcomas. An excellent review of the biology and clinical aspects of sarcoma progression can be found in Part XV of our recently published book on Cancer Metastasis through the Lymphovascular System [110].

## Conclusions and future perspectives

**Stanley P. Leong and Marlys H Witte**

The molecular mechanisms of cancer metastasis through the lymphatic versus blood vessels are still under intense study. In the SLN era, cancer metastasis can be studied in its early stage. Multiple molecules have been found to be associated with the complicated process of metastasis. Thus, blocking these molecules may be potentially adopted as a therapeutic means to control or stop cancer metastasis [76, 111]. New markers and functions of distinct LEC subpopulations in murine skin draining lymph nodes were identified by Fujimoto et al. using single-cell RNA sequencing [81]. A subtype of cortical LEC was identified to be associated with rapid egress of lymphocyte from lymph nodes. These findings of LEC heterogeneity and functions are crucial for future studies relating to the regulation of immune responses by lymph node LECs [81].

Even though we have learned a great deal from recent studies regarding the concept of SLNs and multiple molecules relating to the lymphatic system and trafficking of cancer cells as summarized in this review article, several major questions still are unanswered as:

1. What is the spectrum of cancer heterogeneity?
2. What are the molecular interactions of multiple cell types in the cancer microenvironment to facilitate metastasis?
3. Which biomarkers are used by the cancer clones to metastasize through the lymphatic versus blood vessels that potentially can be used to stratify patients and/ or predict their potential to form metastasis in lymph node and distant sites?

Perhaps, spatial multiplex imaging and single cell gene analysis [112, 113] may be used to tackle the issues of caner heterogeneity and the cancer microenvironment in the future. In the SLN era, it is reasonable to conclude that cancer cells can metastasize through the blood vessels in some cases but in most cases, they seem to use the SLN as the major gateway to enter the circulation for distant metastasis, particularly in melanoma and breast caner. Understanding the precise molecular mechanism of these routes of metastasis by cancer cells is important for the development of effective therapy. It is crucial that clinicians and basic scientists interact closely together to explore the full facets of cancer metastasis to gain success in diagnostic and therapeutic goals.

## Data Availability

No datasets were generated or analysed during the current study.
